# Material Exchange in Photoreceptor Transplantation: Updating Our Understanding of Donor/Host Communication and the Future of Cell Engraftment Science

**DOI:** 10.3389/fncir.2018.00017

**Published:** 2018-03-06

**Authors:** Philip E. B. Nickerson, Arturo Ortin-Martinez, Valerie A. Wallace

**Affiliations:** ^1^Donald K. Johnson Eye Institute, Krembil Research Institute, University Health Network, Toronto, ON, Canada; ^2^Department of Laboratory Medicine and Pathobiology, University of Toronto, Toronto, ON, Canada; ^3^Department of Ophthalmology and Vision Sciences, University of Toronto, Toronto, ON, Canada

**Keywords:** photoreceptors, transplantation, material exchange, retinal degeneration

## Abstract

Considerable research effort has been invested into the transplantation of mammalian photoreceptors into healthy and degenerating mouse eyes. Several platforms of rod and cone fluorescent reporting have been central to refining the isolation, purification and transplantation of photoreceptors. The tracking of engrafted cells, including identifying the position, morphology and degree of donor cell integration post-transplant is highly dependent on the use of fluorescent protein reporters. Improvements in imaging and analysis of transplant recipients have revealed that donor cell fluorescent reporters can transfer into host tissue though a process termed material exchange (ME). This recent discovery has chaperoned a new era of interpretation when reviewing the field’s use of dissociated donor cell preparations, and has prompted scientists to re-examine how we use and interpret the information derived from fluorescence-based tracking tools. In this review, we describe the status of our understanding of ME in photoreceptor transplantation. In addition, we discuss the impact of this discovery on several aspects of historical rod and cone transplantation data, and provide insight into future standards and approaches to advance the field of cell engraftment.

## Introduction to Tissue Regeneration and Cell Replacement Science

The capacity for endogenous repair is effectively absent in the mammalian central nervous system (CNS), which suffers from the marked inability to mobilize stem cell-like repair activity to functionally replace lost neurons following injury, or due to disease (reviewed in Gage and Temple, 2013). Consequently, therapeutic approaches that address neuropathological conditions are relegated to mitigating disease progression, or attenuating the loss of cells through supportive therapies. Cell transplantation is one endeavoring approach to replace lost neurons in CNS tissues. Central to the workflow of pre-clinical cell transplantation is the ability to identify donor cells for procurement, and to identify and track these cells following transplantation into recipient tissues. This is normally achieved through the use of donor cell labeling methodologies that can reveal aspects related to their cell fate, as well as position and morphology post-transplant. Recently, it was discovered that fluorescent reporter signal observed in recipient retinal tissue post-transplant arises due to intercellular movement of donor-derived fluorescent reporters (Pearson et al., 2016; Santos-Ferreira et al., 2016a; Singh et al., 2016; Decembrini et al., 2017; Ortin-Martinez et al., 2017). As reporter and other donor-derived components participate in this process of intercellular material exchange (ME) with host cells, it complicates the interpretation of the efficacy of photoreceptor transplantation. Moreover, the field is now challenged with re-interpreting historical transplantation data in the context of ME, and must consider the possibility that the recovery of visual function in transplanted retinas could be a consequence of this phenomenon, rather than the physical integration of donor cells. This review will address the impact of ME on the experimental workflow of cell transplantation in the eye and will discuss shifting interpretation of historical and future data and new standards of rigorous cell tracking methodologies.

## The Rationale Behind Cell Transplantation in the Eye

**Figure 1:**
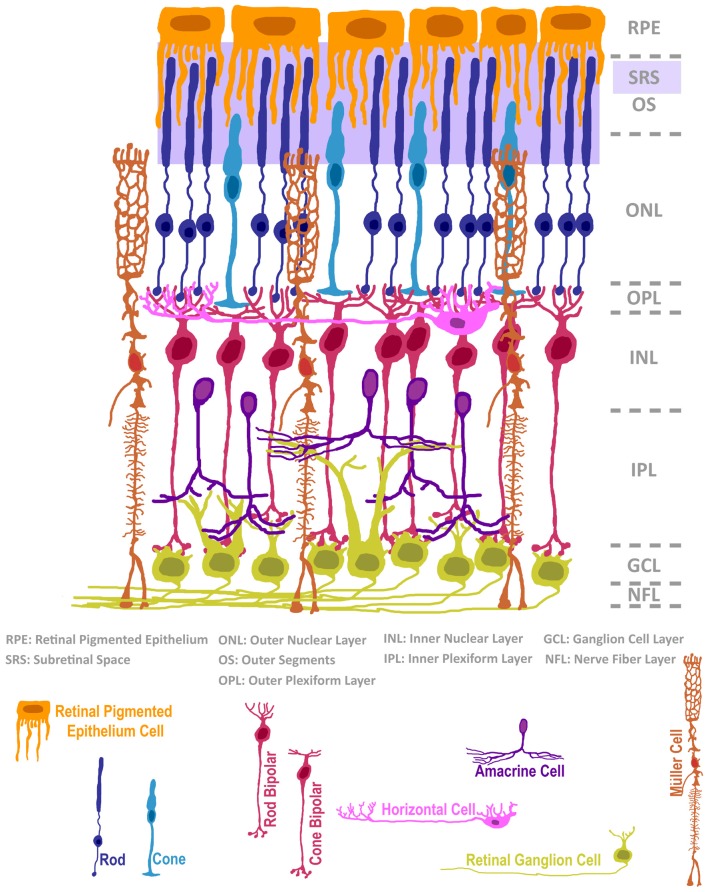
The structure of the mammalian retina, adapted from Ramón y Cajal (1972). The neural retina is composed of seven classes of neurons and a radial glial support cell, located in intermixed strata of nuclear and plexiform layers. The subretinal space (SRS), positioned below the retinal pigmented epithelium, is a surgically accessible domain that is occupied by outer segments (OS) of rods and cones. In cases of retinal degeneration, inner retinal cells, classified as bipolar, horizontal or amacrine interneurons, as well as 3rd order projection ganglion cells, remain largely intact. The Müller radial glial cell is highly relevant in normal retinal homeostasis, and its activity status impacts retinal degeneration and cell transplantation microenvironments.

The experimental workflow of neural cell transplantation encompasses several compartments, including: (i) the isolation or engineering of appropriate cell types; (ii) the safe enrichment or expansion of these cells to generate sufficient numbers for transplant; (iii) the development of surgical delivery protocols; and (iv) the management of cell viability, motility, integration and safe functioning throughout the lifetime of the recipient. Early proof-of-concept neural transplantation experiments in neurodegenerative disease models helped to refine this general workflow. For example, isolation of fetal rat ventral mesencephalon primordium and transplantation into 6-hydroxydopamine-lesioned (Parkinsonian) rats in the 1980’s established that cell replacement could impart some functional recovery in mammals. This work quickly translated into clinical trials in that same decade (Brundin et al., 1986, 1990; reviewed in Trounson and Dewitt, 2016). Although this therapeutic approach has not evolved past early trials, it provided evidence that the human CNS was amenable to neural transplantation, and that animal models could inform human transplant protocols. Adapting established models such as the Parkinson’s model to explore treatment to other areas of the nervous system can be met with challenges. For example, the highly invasive nature of surgical craniotomy and laminectomy procedures can be prohibitive to follow-up evaluation of brain and spinal cord function, motivating science to explore alternative models for CNS testing. As such, when attempting to understand the biology of neural cell transplantation, a more surgically accessible target of CNS tissue would mitigate these challenges. The extracranial anatomy of the eye provides us with minimally-invasive access to CNS tissue, and has served as a tractable beta-test site for exogenous application of cell replacement modeling. In addition to being physically accessible, the microanatomy of the retina and surrounding ocular tissues provides researchers with convenient spatial advantages when developing strategies for cell delivery, and perhaps more importantly, the ability to interpret the efficacy of cell transplantation upon *post-mortem* evaluation (Figure 1). To the first point, delivery of cells to either the vitreous body or into the subretinal space can be achieved via injection through a minor incision, and both sites exhibit some degree of immunoprivilege in response to xenografting (reviewed in Streilein, 2003). Furthermore, the multi-layered nuclear structure of the retina bolsters our ability to identify specific classes of host cells and to contrast this information with the position and morphology of transplanted donor cells. Nuclei of the photoreceptors of the retina, a cell class that mediates the initial photon detection and neural signal transduction in the visual pathway, exclusively occupy the outermost nuclear layer (see diagram in Figure 1). This photoreceptor layer is directly coupled with the subretinal space by rod and cone photoreceptor outer segment (OS) protrusions. This close apposition between a largely monotypic cell layer and a surgically accessible domain offers a condition in which a single class of cell can be theoretically repopulated by donor cell engraftment. Finally, our knowledge of the transcriptional programing that encodes cell fate in the retina, and the library of cell-type-specific markers used to evaluate individual cell types therein is among the most comprehensive in CNS research. For these reasons, much of the general field of neural cell transplantation has benefited from experimentation in the eye.

The goal of clinical cell transplantation is to recover or augment the function of a target organ system such that some therapeutic benefit or cure has been satisfied. Several blinding diseases involve functional disruption of a single class of retinal cell, and in turn, have influenced the direction of cell transplantation research in the eye. Rod and cone photoreceptors are examples of individual retinal cell types that mediate low-light and high acuity color vision, respectively. The loss of rod and cone photoreceptors in conditions such as retinitis pigmentosa (RP) and age-related macular degeneration, respectively, results in progressive blinding in patients that collectively span many age groups. The role of photoreceptors in the initial transduction of light into a neurochemical signal positions these cells at the leading edge of the visual circuitry. The loss of rods or cones can be due to either primary initiation of cell death, the secondary effects brought about by the death of other ocular cell types such as the retinal pigmented epithelium, or the loss of other retinal cells such as the secondary loss of cones following the death of rods (reviewed in Amram et al., 2017). Although current treatment strategies aim to attenuate cell loss in photoreceptor-related pathological conditions, a clinical procedure to replace lost photoreceptors has not been established. Thus, diagnosis of photoreceptor degenerative diseases is accompanied with a prognosis of progressive loss of vision. Although significant advancements have been made in gene therapy and transplantation of retinal pigmented epithelium as avenues to mitigate photoreceptor loss (reviewed in Nommiste et al., 2017; Ovando-Roche et al., 2017), these approaches are not effective in a condition in which rods and cones have already died. Cell replacement therapy, if realized as a generally deployable rod and cone replacement platform, could provide us with the first curative approach to treat blinding disorders that target photoreceptors.

## A Brief History of Cell Tracking in Photoreceptor Transplantation

Early photoreceptor cell transplantation studies focused on testing whether the deposition of cells in proximity to the photoreceptor layer could establish long-term survival and physical integration of donors in the recipient ocular environment and retina. Experiments aimed at investigating whether orthotopically transplanted retinal tissue into brain could regenerate axonal projections into appropriate terminal regions established a primordial work flow for retinal procurement and surgical grafting procedures in mammals (McLoon and Lund, 1980b). Lund’s early work tackled many of the technical hurdles of retinal cell transplantation, including the use of horseradish peroxidase labeling methods for graft visualization (McLoon and Lund, 1980a). Extensions of this protocol were widely used in retinal cell grafting procedures, with additional utility of enzymatic dissociation in both wildtype and the Royal College of Surgeons (RCS) *Mertk* (Vollrath et al., 2001) retinal degeneration rat model (del Cerro et al., 1989; Silverman and Hughes, 1989; Gouras et al., 1991a,b,c; Kwan et al., 1999). Integral to this progression was the ability to visualize the cell deposit, both in living and *post-humous* preparations. Attempts at *in vivo* fundoscopy proved difficult in transplantation science, as it lacked the spatial resolution required to monitor small bolus deposits in rodents (del Cerro et al., 1989). This motivated researchers to re-employ donor pre-labeling strategies such as Fast Blue and the carbocyanine dye DiI to visualize cells in histological sections (del Cerro et al., 1989; Gouras et al., 1991a,b). During that same era, the mixing of RPE and photoreceptors served as a pigment contrasting reagent in co-transplantation experiments for the identification of the injected bolus (Silverman and Hughes, 1989). The identification of rod photoreceptors within these pigment-contrasted structures was achieved by morphological evaluation of nuclear euchromatin/heterochromatin structure using histological stains (Gouras et al., 1991b). A more precise evolution in donor cell identification emerged with the testing of thymidine-analog incorporation (Gouras et al., 1991a). In contexts in which donor cells could be targeted during their birth, tritiated thymidine (3H-thymidine) or 5-bromo-2′-deoxyuridine (BrdU) provided an indelible marker of donor cell DNA. By extension, donor cell nuclei and evidence of nuclear migration or translocation could be evaluated using this method. Although a significant advance, thymidine-analog labeling has some caveats, including signal transfer from dead cells to dividing progenitors, which can generate false-positives (Burns et al., 2006). It is also uncertain whether this method imparts some change in the inherent engraftment ability of donors, although no clear precedent for this exists in the literature. Moreover, the nuclear localization of thymidine-analogs does not provide data on the morphology of cell soma and somatic processes. Testing of early cytoplasmic-localized LacZ reporter mice as donors enhanced the visualization of cell morphology post-grafting (Gouras et al., 1991c). Of note, side-by-side comparison of the thymidine-analog nuclear pre-labeling method with NSE-LacZ reporter prompted the conclusion that multiple cell tracking (nuclear + cytoplasmic) methodologies should be co-deployed to control for weaknesses of each method (Seiler and Aramant, 1995). Although cytoplasmic reporter is useful in morphological interpretation, nuclear labeling is required to identify the central positioning of the cell within the recipient tissue. It was when this combinatorial method was revisited that ME was discovered.

In addition to cell pre-labeling techniques, the clever use of immunocytochemical labeling complemented these approaches. For example, immunodetection of Opsins in intact donor photoreceptors that had been grafted into highly degenerated recipients identified donors in contrast to hosts that were largely devoid of constitutive Opsin staining (Silverman and Hughes, 1989). Furthermore, xenografting and co-culture studies that employed the earlier iterations of induced human photoreceptors utilized anti-Human nuclear antigen immunocytochemistry to resolve donor (human) and recipient (rodent) cell populations (for examples, see Lamba et al., 2006, 2009; Barnea-Cramer et al., 2016). Although highly specific, this method cannot be used in allografting studies. Collectively, these pioneering achievements of identifying donor cells post-grafting consolidated the paradigm of cell tracing in therapeutic models of cell replacement, and formed a basis for future donor cell detection methods.

Further modification of detection protocols would be needed to adapt to emerging issues in cell engraftment science, including the prospective enrichment of specific classes of retinal cells such as rods and cones. The synthesis or adaptation of gene encoded fluorescent reporters was critical to the establishment of modernized photoreceptor cell isolation and purification protocols. Early utilization of virus infection to mark donor cells demonstrated that ubiquitous expression vectors for GFP permit detection of donor cells (Kicic et al., 2003). Although highly relevant by today’s standards, the use of lenti- and adenovirus pre-labeling imposes added technical strain on the complex transplant workflow, including the demand for infection controls and the problem of low cell numbers. Formative studies from the Swaroop lab developed a rod-specific photoreceptor transgenic reporter strain that utilized the *Neural Retina Leucine Zipper* promoter to drive green fluorescent protein (*NRL-GFP*; Akimoto et al., 2006). GFP has the advantage over other cytoplamic markers, like lacZ, as it does not require fixation and staining for detection and can therefore be used for prospective enrichment using fluorescent activated cell sorting. Furthermore, histological evaluation of GFP in transplants is more technically amenable than lacZ, and permits the inclusion of multiple fluorescent markers. Finally, relative to viral delivery of GFP, the NRL-GFP lineage reporter provides complete coverage of the rod population, and avoids confounds associated with viral infection. Adaptation of this mouse reporter and accompanied retinal dissociation protocols, to intraocular transplantation demonstrated that GFP-expressing rod precursor cells could be grafted into the subretinal space of recipient mice (MacLaren et al., 2006). This work demonstrated that GFP pre-labeled rod cells persist in the subretinal space for weeks, recapitulating earlier observations in rats (Kicic et al., 2003). Remarkably, an additional phenotype was observed, wherein GFP-positive cells were observed in the adjacent, recipient outer nuclear layer (ONL) where resident host photoreceptors persist (MacLaren et al., 2006). The morphology of these GFP-positive cells was initially interpreted as evidence that transplanted rod precursors could migrate to appropriate positions and elaborate apical and basal elements, such as OS and terminal synaptic structures, respectively. As such, the term *integration* was used to refer to GFP-positive signal in the ONL that bore this morphological resemblance to constitutive photoreceptors. When transplanted into the *Rho*^(−/−)^ (blind) mouse model, *NRL-GFP* cells could impart photoresponsiveness to the retina, as measured by improvements in electroretinogram profiles and re-establishment of pupillary responses (MacLaren et al., 2006). Collectively, these seminal data that were reliant on novel GFP reporter methods provided the proof-of-principle that cell replacement in the mammalian retina was feasible, and furthermore, had the potential to provide functional improvement in blind recipients. More recent refinement of this protocol demonstrated improvement in visually-guided behaviors and visual cortex activity (Pearson et al., 2012). Moreover, this grafting method was most efficient when using a post-mitotic precursor cell population (MacLaren et al., 2006), contradicting the popular prediction that efficacious photoreceptor replacement would occur by transplanting immature, dividing retinal progenitor cells that would respond to environmental cues and adopt the fate of the missing cell type. As the paracrine signaling interplay between donor and host cell types is presumed to be variable according to the specific disease state present, donor cell functional plasticity in response to these cues has been regarded as a fundamental asset in donor cell preparation.

Since 2006, a myriad of publications have described variations on this engraftment protocol, including those which address the replacement of cone cell photoreceptors (Santos-Ferreira et al., 2015; Smiley et al., 2016; Decembrini et al., 2017; Gonzalez-Cordero et al., 2017) the supportive retinal pigmented epithelium (reviewed in Amram et al., 2017), or ocular cells generated from cultured adult retinal stem cells (Clarke et al., 2012). Variations on the source and age of donor cell (i.e., primary embryonic retinal progenitor, postnatal and adult retinal tissue; embryonic and induced stem cell-derived retinal, and non-neural) and the genetic background of blind and immunocompromised recipients have been assayed (reviewed in Santos-Ferreira T. F. et al., 2016). One unifying element of the field during this time has been the utility of fluorescent reporter donors, such as the *NRL-GFP* mouse as a means to track the position, morphology, and fate of transplanted rod photoreceptor cells (reviewed in Boudreau-Pinsonneault and Cayouette, 2018). Several groups have excelled at providing increasingly-resolved imaging of donor cells in the bolus in the subretinal space of grafted mice, and of integrated GFP cells located in the ONL (herein referred to as ONL-GFP cells). More formal attempts at correlating the number, position, and morphology of ONL-GFP cells with the degree of functional recovery hinted that greater numbers of ONL-GFP cells correspond to greater visual recovery (Barber et al., 2013; Warre-Cornish et al., 2014; Ballios et al., 2015). The observation of ONL-GFP cells, and further electron microscopic analysis of their synaptic contacts with host tissue presented an interpretation of functional integration that was predicated on the assumption that the GFP marker used to identify those cells remains in donor cells. Some brief attempts to prove the reliability of GFP as a donor cell marker using thymidine-analog pre-labeling of donor DNA, and a two color (CFP/GFP) donor/host transplantation scheme hinted that donor cell integration can occur, and that donor cell nuclei migrate into recipient tissue (MacLaren et al., 2006; Bartsch et al., 2008). Given the paucity of evidence describing the intercellular transfer of cytoplasmic GFP in other mammalian systems, the photoreceptor transplant field proceeded for more than a decade without adequate and rigorous testing of this assumption.

## The Discovery of Donor/Host Material Exchange (ME): A Cone’s Perspective

The natural evolution of photoreceptor transplantation research, and the development of cone transplantation protocols provided researchers with increasingly novel insight into the fate of transplanted cells. Expanding on the rod engraftment workflow reported in MacLaren et al. (2006), Lakowski et al. (2010) enriched rod and cone cells with the pan-photoreceptor reporter, *Crx-GFP* from various ages and determined that early embryonic preparations generated engraftable, Rxrγ-expressing cone cells. Following transplant, these cells generally phenocopied the ONL-GFP integration observed using *NRL-GFP* donors (MacLaren et al., 2006), with the additional contrast of cone marker co-labeling in a subset of integrated GFP cells. Work from the Ader group then provided the first demonstration of prospective cone cell enrichment using a combination of genetic, fluorescent reporter, and cell surface selection methods (Santos-Ferreira et al., 2015). Specifically, they utilized *NRL*^(−/−)^ mutant donors in which all rod cells fail to specify and undergo a default fate switch to a hybrid, cone-like cell (Mears et al., 2001; Yoshida et al., 2004). Crossing these mutants with actin-GFP reporter mutants, and enrichment of photoreceptors using CD73 magnetic beads generated GFP-expressing hybrid-cone donors (Santos-Ferreira et al., 2015). Transplanted, *NRL*^(−/−)^ :*actin-GFP* donors resulted in ONL-GFP labeling in recipients that bore the morphological resemblance to rod and cone cells. Furthermore, a subset of ONL-GFP cells stained for cone arrestin, adding credence to the notion that donor cone cells successfully transplant and retain their neurochemical attributes. In March 2016, our group published the first example of FACS-enrichment and transplantation of GFP-positive, endogenous cones from postnatal mice (Smiley et al., 2016). Our approach employed the use of a novel *Coiled-Coil Domain Containing 136* *(Ccdc136)* GFP-trapped allele reporter mouse that expresses GFP in developing and adult cones, as well as adult bipolar neurons. In addition to transplanting postnatal GFP-expressing cones from a wildtype background, *Ccdc136-GFP* mice were similarly crossed with *NRL*^(−/−)^ (hybrid cone-only) mutants to generate high numbers of transplantable, hybrid-cone cells. In either case, transplantation of postnatal *Ccdc-GFP* or *NRL*^(−/−)^:*Ccdc-GFP*-hybrid-cones into a rod-dominant (wildtype) recipient resulted in ONL-GFP labeling. This approach also provided additional contrast between donor and host cells, as the nuclear architecture of cone cells appears as larger, elongated and multifocal heterochromatin structures. This differs from the smaller, round and single chromocenter nuclear morphology that is a hallmark feature of the euchromatin/heterochromatin inversion present in rods (Solovei et al., 2009). Following cone-GFP transplantation into wildtype recipients, we observed what appeared to be GFP labeling in host rods, based on nuclear morphology. This was effectively a heterotypic transplant experiment that produced a homotypic result, raising the question of whether cone donor cells undergo a fate switch to become rods post-grafting or that this represented some type of fusion event. Later that year and in 2017, five groups (including ours) working independently, published articles that collectively described the detection of GFP signal and other donor photoreceptor material in host cells post-transplant (Pearson et al., 2016; Santos-Ferreira et al., 2016a; Singh et al., 2016; Decembrini et al., 2017; Ortin-Martinez et al., 2017). The implications of these findings, described in detail below, have greatly shifted the interpretation of historical data in the field, and offer a novel topic of donor/host intercellular communication via ME.

The convincing demonstration of ME between donor and host photoreceptors involved multiple methodologies aimed at buttressing the researcher’s ability to resolve donor vs. host cell populations (Summarized in Table 1). Common to all five publications was the use of two-color (donor vs. host) fluorescence reporter mice and the detection of fluorescence co-localization post-transplant. Although employed in earlier publications as a method to disprove donor/host cellular fusion (MacLaren et al., 2006; Bartsch et al., 2008), the data presented did not include quantification or multiple examples of single-labeled ONL-GFP cells. Imaging of tightly packed cells of the retina can be technically challenging, and revisiting this technique with modernized analytic approaches and instrumentation challenged these earlier results. With our group, the use of a Zeiss LSM780 confocal microscope provided us with broad freedom to control laser light power, use precise and sensitive spectral detection, and employ spectral unmixing algorithms to more easily and precisely interpret recipient tissues. All groups employed the use of *NRL-GFP* mice to identify rod cells, and a ubiquitous red fluorescent reporter as a contrasting reagent. In one case, the red reporter was a membrane-tethered variant (Ortin-Martinez et al., 2017). Furthermore, *NRL-GFP* was used as both a donor (Pearson et al., 2016; Santos-Ferreira et al., 2016a; Singh et al., 2016) and host (Ortin-Martinez et al., 2017) reference. In all cases, confocal microscopic imaging identified the presence of double-labeled cells in both the host ONL, and in the donor bolus, indicating that fluorophore transfer is bi-directional. These results were corroborated and quantified by flow cytometry of dissociated recipient retinas, which is more sensitive at detecting double labeled cells (Basiji et al., 2007; Pearson et al., 2016; Santos-Ferreira et al., 2016a). As a complementary approach, all groups utilized one or multiple forms of donor cell nuclear identification. Pre-labeling of donor cells with the thymidine-analog EdU (Santos-Ferreira et al., 2016a; Ortin-Martinez et al., 2017) revealed that donor cell nuclei failed to migrate into the ONL and remained in subretinal space. These results were supported by studies in which sex-mismatched donor/host transplants resulted in the non-overlap of male and female nuclei, as identified by sex chromosome fluorescence *in situ* hybridization (Pearson et al., 2016; Santos-Ferreira et al., 2016a; Singh et al., 2016). A third approach to resolve donor and host cells was to perform transplants with either rod or cone donors into recipients that are either rod (wildtype) or cone (*NRL*^(−/−)^)-dominant, and then compare the expected fate of donor cells with the observed fate of ONL-GFP cells post-transplant. By transplanting rod-GFP cells into the cone-dominant recipient, cone-GFP cells into the rod-dominant, and complementary controls, it was determined that GFP labeling in the recipient retina corresponded to the dominant cell type within that recipient (Ortin-Martinez et al., 2017). This, in conjunction with the assessment of cell fate by nuclear morphometry and EdU pre-labeling supported the conclusion that donor cells transfer their fluorescent reporter signal to host cells, with no evidence of rod/cone trans-fating post-transplant.

**Table 1:** Summary of techniques used to identify material exchange.

		Ortin-Martinez	Pearson	Santos-Ferreira	Singh	Decembrini
**DONOR CELL TYPE(S) or BACKGROUND**		Rod precursors	Rod precursors	Rod precursors	Rod precursors	Cone precursors	
		Cone precursors	e11 progenitors	BL/6j	Rd1	
		NRL^(−/−)^ hybrid- cone precursors	ES-derived PRs		BL/6j	
			Fibroblasts				
**RECIPIENT MODEL BACKGROUND(S)**		BL/6j	Prph2^(rd1/Rd1)^	BL/6j	BL/6j	BL/6j
		Nrl^(−/−)^	BL/6j	B2-Cre	Crx-Cre	
		Crx^(−/−)^	Gnat1^(−/−)^			
**METHOD OF DONOR/HOST RESOLUTION**	**Two-color fluorescence**	**Y**	**Y**	**Y**	**Y**	**Y**	
	**EdU pre-labeling**	**Y**	**-**	**Y**	**-**	**-**	
	**Y-chromosome FISH**	**-**	**Y**	**Y**	**Y**	**-**	
	**Donor/Host fate mismatch**	**Y**	**-**	**-**	**-**	**-**	
	**Cre-recombinase reporter**	**-**	**Y**	**Y**	**Y**	**-**	
	**Ectopic photoreceptor protein (Cone Arrestin or rod α transducin)**	**Y**	**Y**	**-**	**-**	**-**	
**NOTABLE OBSERVATIONS**		- Transfer of GFP to rods, cones and cods, with 1000-fold higher exchange in an Nrl^(−/−)^ background. - Bidirectional (donor/host) and second order transfer to INL cells.	- Bidirectional material exchange between donor and host rods - eGFP uptake does not involve free-proteins and cannot be elicited by fibroblast donors.	- Material transfer from host to donor rods.	- Bidirectional material exchange between donor and host rods - Transfer as early as 3 days post-transplant.	- Cone to rod material transfer

Although much of these data propose that GFP participates in some form of intercellular motility, several issues remain such as identifying the exchange mechanism. Close evaluation of GFP-labeled cells failed to identify binucleated events, arguing that classical cell fusion into a polyploid hybrid can be excluded from the list of candidate mechanisms. Furthermore, whether the GFP signal moves in its protein form or by nucleotide (RNA/DNA) encoding and expression in host photoreceptors has yet to be resolved. We do know, however, that immunocytochemical profiling of photoreceptor-related proteins identified that ME extends beyond that of GFP, with cone arrestin being transferred from *NRL*^(−/−)^ host cones to *NRL-GFP* rod donors (Ortin-Martinez et al., 2017). Indeed, the original Maclaren article demonstrated Peripherin-2 and rhodopsin localization in ONL-GFP cells of *Prph2^rd2/rd2^* and *Rho*^(−/−)^ recipients following NRL-GFP transplantation. This observation is consistent with subsequent reports of rod α-transducin and Peripherin-2 localization following rod transplantation into *Gnat1*^(−/−)^ and *Prph2^rd2/rd2^* recipients, respectively (Pearson et al., 2012; Barber et al., 2013; Gonzalez-Cordero et al., 2013; discussed in Pearson et al., 2016). The demonstration of ME involving the membrane-tethered tdTomato reporter (Ortin-Martinez et al., 2017) is further evidence that this phenomenon is not restricted to cytoplasmic material. The demonstration that nuclear-targeted material can also engage in ME was provided by experiments that utilized Cre recombinase, to elicit *loxp-STOP-loxp* excision and fluorescence reporter expression in transplant counterparts. Specifically, transplantation of Cre-expressing donors into Cre-sensitive, dsRed reporter recipients resulted in reporter expression in recipient photoreceptors (Pearson et al., 2016). The expression of Cre in donor cells has been achieved through both transgenic mutant (Wallace, unpublished) and virally transduced (Pearson et al., 2016) donors with appropriate controls. Reciprocal experiments wherein Cre-sensitive reporter donors were transplanted into Cre-expressing hosts also demonstrated reporter activation (Santos-Ferreira et al., 2016a; Singh et al., 2016). Interestingly, Cre-induced fluorescent reporter also engages in ME back to Cre-expressing cells in what can be described as a reverberating event, indicating that some level of persistent intercellular communication remains intact (Singh et al., 2016). Carefully designed experiments that employ timed induction of Cre expression and pulse-chase analysis will be required to detail the kinetics and saliency of these communicative associations.

**Figure 2:**
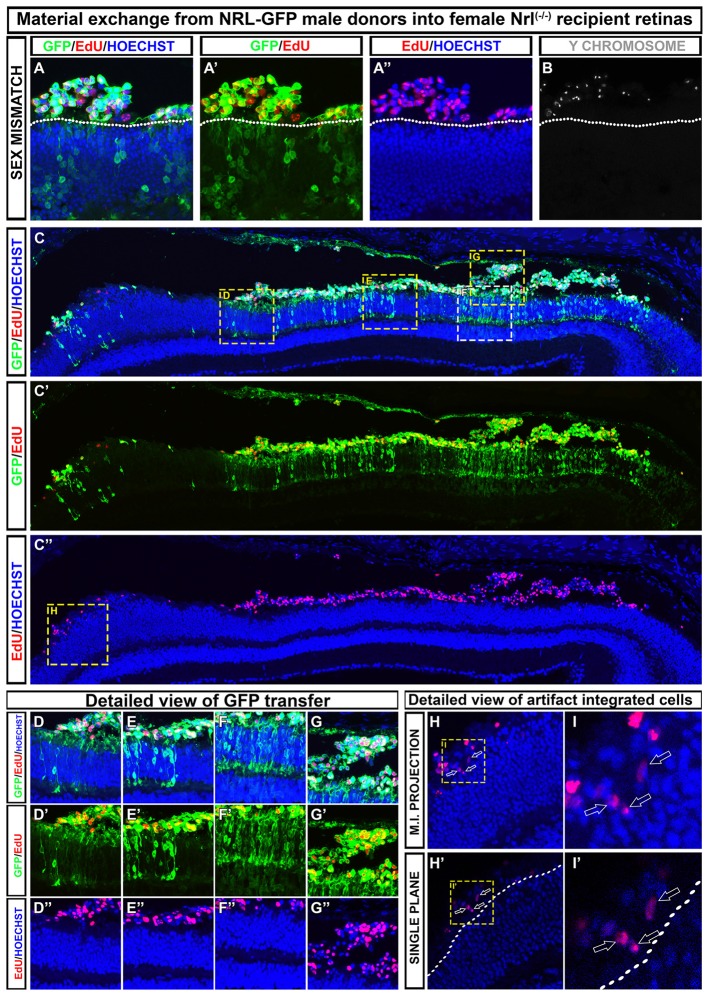
Examples of how to discriminate between material exchange (ME) and cell integration post-transplant. A representative section adapted from Ortin-Martinez et al. (2017). **(A–B)** Transplanted EdU pre-labeled, NRL-GFP expressing male (Y-chromosome FISH-positive) donors that are exclusively localized to the subretinal space of *NRL*^(−/−)^ recipients. Outer nuclear layer (ONL)-GFP labeling is robust in the recipient, highlighting the robustness of cytoplasmic ME. **(C–C″)** Low magnification view of a retinal section showing regional variability in donor and host signal patterning at various points along the retina. **(D–G″)** Insets from **(C)** that demonstrate the variable degree of ONL-GFP signal observed in a single retinal section. **(H–I′)** Maximum intensity projection **(H,I)** rendering of EdU pre-labeled donor cells gives the impression of cell integration into the recipient ONL. Single optical sectioning **(H′–I′)**, however, shows that the EdU+ donor cell is apical to the host ONL and, therefore, not integrated.

Differences in how various research groups interpret cell integration is evident in recent literature (Pearson et al., 2016; Santos-Ferreira et al., 2016a; Singh et al., 2016; Decembrini et al., 2017; Ortin-Martinez et al., 2017), and thus, there is no rock-solid consensus on the true integration rate of donor cells into recipient tissue. Although some have concluded that a small number of truly integrated cells were quantifiable by both histological and flow cytometric methodologies, the small number of events reported make it very important that these findings be replicated in other labs. We have compared various imaging and image processing approaches in the context of dual-fluorescence, EdU, and Y-chromosome FISH assays in an effort to address this issue. In Figure 2, we provide representative examples of the types of data generated using these techniques, and which are similarly reported in Ortin-Martinez et al. (2017). In all examples, we conclude that there are no donor cells that occupy positions within the retina proper when transplanting *NRL-GFP* donors into the *NRL*^(−/−)^ recipient. Critical to this conclusion is the rendering of images as single optical plane confocal micrographs. In Figures 2H–I′, misinterpretation of cell position is imparted by the use of maximum intensity projection of z-axis scans. Single plane evaluation reveals a non-integrated location of more apical donor cells that more accurately reflects the subtle microanatomy of the apical ONL.

## Material Exchange Requires Photoreceptor-To-Photoreceptor Communication

It is important to understand the mechanism that mediates ME, and what factors affect the efficiency of this process. Historical data of GFP labeling in the host ONL following transplant provides us with a retrospective lens through which we can identify the contextual restrictions of donor/host ME. We know that donor cell type, age, and source collectively impart the competence for ME (Summarized in Table 1). For example, postnatal day 4–6 rod precursors exhibit the highest competence for GFP transfer, with declining transfer being observed in younger and older donors (MacLaren et al., 2006). Work from the Reh lab, however, demonstrated equal GFP labeling capacity when comparing postnatal day 4–6 and postnatal day 79 donors (Gust and Reh, 2011). This effect was dependent on the persistent observation of cells in the subretinal space at the time of evaluation, raising the possibility that the success of adult donors is a function of donor age-dependent differences in photoreceptor viability. Moreover, GFP transfer appears to be unique to photoreceptors as transplants of GFP labeled retinal progenitor cells (MacLaren et al., 2006), hippocampal neural progenitor cells (Guo et al., 2003) or fibroblasts (Pearson et al., 2016) does not result in GFP labeling in photoreceptors in the recipient retina. There is also some evidence that ME involves close physical association between donor and host photoreceptors, as the majority of GFP-labeled cells in rod-grafted Nrl^(−/−)^ retinas are associated with the presence of an adjacent, apically located donor cell (Ortin-Martinez et al., 2017). However, the requirement for donor-host association is not absolute, as it has been suggested that GFP-labeled cells in the host can be located at a distance from donor cells (Pearson et al., 2016). Finally, the exchange could be transient, as GFP labeling disappears following the death of donor cells in the subretinal space (West et al., 2010). Perhaps more interesting and informative is the highly variable degree to which ME is active across the spectrum of recipient models tested. Lower efficiency in ME is observed in the wildtype eye, which is dominated by rods and exhibits only minimal structural pathology following resolution of surgical detachment following transplant (MacLaren et al., 2006). Disruption of the outer limiting membrane (OLM) in wildtype retinas by treatment with alpha-aminoadipic acid (AAA) or ZO-1 knockdown, however, increases the amount of ME observed by 2-3-fold, suggesting that the OLM or associated physiological processes following AAA treatment, such as reactive gliosis, inhibit ME (West et al., 2008; Pearson et al., 2010). To the contrary, evidence that reactive gliosis *per se* generally inhibits ME comes from observations that retinal degeneration models in the absence of AAA treatment exhibit an inverse correlation between gliosis and ONL-GFP labeling (Barber et al., 2013). Transplantation of either rods, cones, or *NRL*^(−/−)^ hybrid cones into the *NRL*^(−/−)^ recipient results in 100 to 1000-fold increase in ME (Ortin-Martinez et al., 2017). Several candidate factors in the *NRL*^(−/−)^ recipient platform have been proposed to contribute to this marked increase. We reported that focal sites of ONL-GFP were coincident with perforations in the mutant OLM, observed by disruptions in the junctional protein ZO-1 immunofluorescence signal and 3D rendering of confocal micrographs (Ortin-Martinez et al., 2017). Although an increase in ME accompanying OLM disruption is consistent with previous observations, the vastly increased degree of ME observed in *NRL*^(−/−)^ recipients in comparison suggests that additional (multiple) factors can impact this phenomenon. It is known that *NRL*^(−/−)^ retinas harbor some unique phenotypes including the fate switch of all rods to a hybrid cone default, the progressive formation of structural rosetting due to disruption in the ONL, and the failure of OS to mature as their wildtype counterparts (Daniele et al., 2005; Stuck et al., 2012). Thus, multiple factors exist as reasonable candidates that can contribute to the competence of ME.

## 2nd Order Transfer and Candidate Mechanisms of Intercellular Material Exchange

The robustness of GFP signal observed in recipient retinas following ME has been variable across recipient platforms. For example, we determined that the evaluation of wildtype recipient ME using confocal microscopy requires much higher laser excitation and detector gain levels when compared to the highly robust signal observed in *NRL*^(−/−)^ recipients (Ortin-Martinez et al., 2017). We hypothesize that this reflects the variable amounts of GFP participating in ME, rather than phenomenon related to fluorophore quantum efficiency. In either case, the detection and evaluation of GFP signal in recipient cells requires adjustments in instrument sensitivity as one changes the combination of donor/host tissues. In an attempt to standardize these detection settings across donor/host combinations, we discovered the presence of low-level GFP signal in what appeared to be in downstream cells located in the inner nuclear layer (INL) of *NRL*^(−/−)^ recipients following NRL-GFP donor cell transplantation (Ortin-Martinez et al., 2017). Using stringent negative control standards to ensure imaging parameters that exclude background signal, follow-up immunocytochemical profiling identified that GFP could be observed in bipolar neurons and Müller glia of these recipients, and not in horizontal cells. Using the same approach, we determined that this GFP signal is not present in microglia, suggesting that INL-GFP does not emerge by classical phagocytic activity that is inherent to surgically damaged retinas. We then evaluated NRL-GFP (surgically naïve control) retinas to determine whether INL-GFP is specific to the surgical deposition to NRL-GFP donor cells, or whether it is also a property of constitutive NRL-GFP expression by rod photoreceptors. In NRL-GFP, unmanipulated (control) retinas, this same pattern of low-level GFP signal in the INL was evident, suggesting that intercellular exchange could be a constitutive property of the mammalian retina. What is difficult to reconcile with this observation is whether there is a role for synaptic coupling, and thus classical 2nd order circuit transfer from rods to bipolar cells in the observed INL-GFP signal. Although GFP labeling of bipolar cells with a rod-driven GFP reporter favors the idea of synaptic coupling, the presence of labeled Müller glia challenges this notion. Furthermore, the use of Cre reporter mutants in donor/host ME evaluation has not demonstrated recombination in inner retinal cells (Pearson et al., 2016; Santos-Ferreira et al., 2016a; Singh et al., 2016). This could be explained by differences in reporter production, as *NRL-GFP* mice produce very high amounts of GFP, whereas many Cre platforms such as the *Crx-Cre* exhibit comparatively lower levels of Cre (Wallace, personal observations). It is conceivable that *NRL-GFP* rods, for example, are attempting to manage the high GFP load by recruiting lysosomal or excretory pathways. Similarly, the passive incorporation of GFP into normal management compartments such as the maturing photoreceptor endosomal pathway could result in motile GFP arrangements. Recent observations described in *Drosophila* identify intercellular motility of Delta protein between developing myoblasts and air sac cells, and Wingless between wing disc cells and myoblasts (Huang and Kornberg, 2015). This motility is mediated by the filopodia-like structures termed cytonemes which have been demonstrated to direct morphogenic events during fly development (reviewed in Kornberg, 2017). Thus, a number of probable candidates exist that could mediate both donor/host and constitutive photoreceptor/bipolar cell intercellular communication. In light of these details, a more thorough concise and definitive method for evaluating 2nd order transfer needs to be formulated that will likely involve the use of circuit tracing methods as a contrasting reagent for synaptic and gap junction coupling and electrophysiology. For example, the use of the trans-synaptic viral (vesicular stomatitis or rabies) trans-synaptic GFP complementation, or barley lectin tracing by a rod cell-driven system would help address this question. As structures such as cytonemes and tunneling nanotubes rarely survive fixation and tissue processing procedures, the incorporation of live imaging and fusion reporters are likely methodologies that would illuminate the kinetics of cell-cell contact and substrate delivery.

## Standards for Distinguishing Cell Integration vs. Material Exchange

Clearly resolving ME from *bona fide* cell integration remains an important challenge in the field of cell replacement modeling, and it would be prudent that a minimum set of standards be established that would legitimize novel data in photoreceptor transplantation. The early standard of combining donor nuclear identification (i.e., thymidine analog) with cytoplasmic reporter (i.e., lacZ or GFP) labeling would re-establish a reliable standard for characterizing cell position and morphology. EdU, BrdU, CldU or IdU pre-labeling offer economical solutions to donor nuclear identification, and can all be complemented by sex-mismatched Y chromosome FISH. Species-specific markers, such as anti-human nuclear antigen immunolabeling in human xenografts, would also provide an adjunct to EdU or Y chromosome FISH, with the caution that nuclear proteins can participate in ME. Utilization of these techniques would form a minimum standard until more modernized reagents are formulated that address functional connectivity and circuit participation.

## The Impact of Material Exchange on the Restoration of Visual Function Following Photoreceptor Transplantation

**Figure 3:**
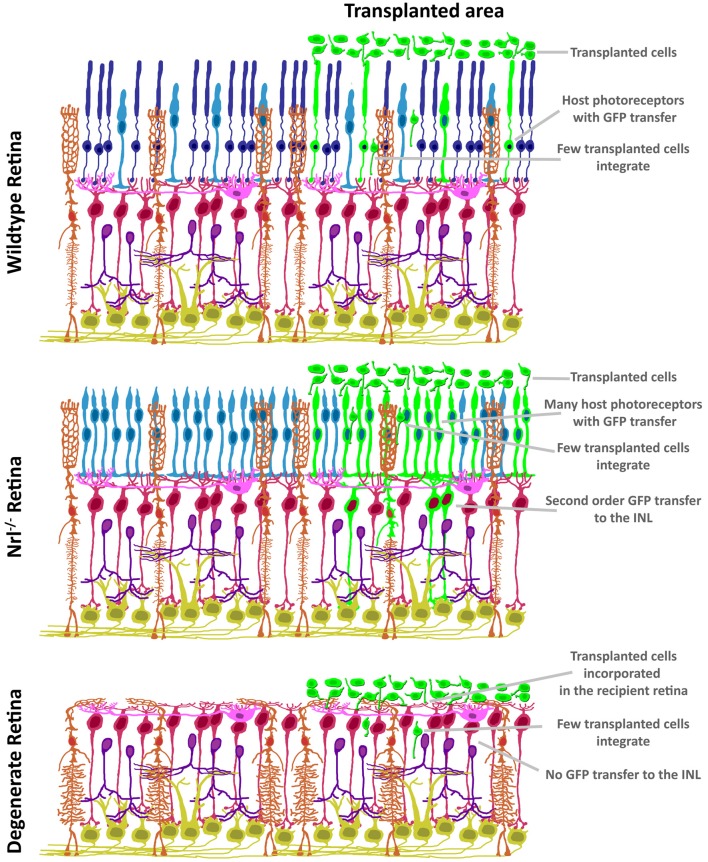
Variability in GFP patterning is dependent on mutant background. In contrast to the restricted ONL-GFP signal observed in wildtype background at 21 days following transplant (top), the *NRL*^(−/−)^ hybrid-cone only recipient (middle) exhibits robust GFP signal that extends to Müller glia and bipolar neurons. In contrast, no GFP ME has been reported in the degenerating retina (bottom), suggesting a photoreceptor-to-photoreceptor modality of intercellular communication.

The discovery of ME raises concerns over the impact of donor/host intercellular communication in the pre-clinical setting. As summarized in Figure 3, both the mutant background and degenerative state of the recipient ocular environment dramatically impact the degree and distribution of ME. ME appears to require photoreceptors in the recipient, as transfer to the INL has not been reported in mouse models with complete ONL degeneration. Many induced and mutant platforms exist that mimic either the failure of photoreceptors to specify/differentiate, or the progressive loss of photoreceptors over time as analogs to blinding disease states. What is very intriguing is the disruption of normal ME kinetics in the presence of ONL thinning. The relationship between donor cells and host retina at early stages of photoreceptor loss is very different from that observed in the retinas in which the ONL is completely denuded. As such, the natural transition from proof-of-concept to pre-clinical modeling in photoreceptor transplantation has been guided by available animal models of retinal degeneration. Transplantation of cones (Pearson et al., 2010; Santos-Ferreira et al., 2015; Smiley et al., 2016; Gonzalez-Cordero et al., 2017; Kruczek et al., 2017), rods (MacLaren et al., 2006; Pearson et al., 2012; Barber et al., 2013; Homma et al., 2013; Singh et al., 2013; Santos-Ferreira et al., 2016b; Wang et al., 2016; Ortin-Martinez et al., 2017), or both together (Lamba et al., 2009; Lakowski et al., 2010; Tucker et al., 2011; Barnea-Cramer et al., 2016; Mandai et al., 2017; Zhu et al., 2017) has been reported, and addresses the goal of establishing a vision restoration strategy in experimental models of human retinal diseases. Several recipient animal models of blinding pathologies in humans have been used as transplant recipients (Table 2). Efforts to assay these diverse pathological microenvironments as well as disease progression at various recipient ages has provided valuable insight into how transplanted donor cells respond to ectopic engraftment conditions.

**Table 2:** Recipient models used in photoreceptor transplantation modeling.

Human disease	Mutant animal	Reference
Macular dystrophy	*Prom1*^(−/−)^	Santos-Ferreira et al. (2016b)
Retinitis pigmentosa (RP)	*S334ter-3 (rhodopsin)*	Seiler et al. (2010)
	*P23H (rhodopsin)*	Yang et al. (2010)
	*P23H* pigs *(rhodopsin)*	Wang et al. (2016)
	*PDE6β*^(rd1/rd1)^	Barber et al. (2013), Singh et al. (2013), Barnea-Cramer et al. (2016) and Mandai et al. (2017)
	*Prph2*^(+/^^Δ^^307)^	Barber et al. (2013)
	*Prph2*^(rd2/rd2)^	Barber et al. (2013)
	*Rho*^(−/−)^	MacLaren et al. (2006), Tucker et al. (2011) and Barber et al. (2013)
	Cpfl1:Rho^(−/−)^	Santos-Ferreira et al. (2016b)
Leber congenital amaurosis	*Aipl*^(−/−)^	Gonzalez-Cordero et al. (2017) and Kruczek et al. (2017)
	*Crb1*^(rd8/rd8)^	Lakowski et al. (2010) and Barber et al. (2013)
	*Gucy2e*^(−/−)^	Lakowski et al. (2010)
	*Crx*^(−/−)^	Lamba et al. (2009), Homma et al. (2013), Smiley et al. (2016) and Ortin-Martinez et al. (2017)
	*Crxtvrm65*	Zhu et al. (2017)
Achromatopsia	*Cpfl1*	Santos-Ferreira et al. (2015)
Stationary night-blindness	*Gnat1*^(−/−)^	Pearson et al. (2012) and Barber et al. (2013)

## Final Remarks

Taken together, the field has produced evidence using a number of techniques that support the conclusion that photoreceptor transplantation can impart measurable improvements in vision in the context of retinal degeneration. However, the mechanism that mediates this phenotype remains unresolved, and could involve synaptic connectivity, ME, or both. Although not an envisaged therapeutic goal of photoreceptor transplant science, the supportive effects of ME in retinal degeneration could persevere as a legitimate therapeutic modality. Recent demonstration of an indirect mechanism of cone rescue after rod transplantation in a model of RP hints at the efficacy of ME, as grafted rods in the subretinal space appear to restore glucose transport, thus reactivating dormant cones (Wang et al., 2016). With this in mind, the observed putative exchange of material, such as that observed with donor rhodopsin could explain vision rescue reported in blind mice (MacLaren et al., 2006). With continued work in degenerating mutants, it is conceivable that we will conclude that the role of transplanted donors is more pleiotropic in nature, acting via mechanisms that provide metabolic and trophic support that delay the progression of retinal degeneration. Furthermore, synaptic connections between donor and host may not require the physical positioning of donor nuclei within the recipient tissue proper. Thus, the adaptation of connectome reagents to capture the functional contribution of donor photoreceptors will be key to dissecting the context that underlies successful re-establishment of photoreceptive retinal circuits. If realized, the resolution of these questions will provide direction in the field, and allow the refinement of current and development of novel transplant approaches. It is also clear that more work is needed to understand the mechanistic basis of ME in the context of safety, and to assign strong effort to determining the negative implications of this phenomenon in the therapeutic domain. No formal work has been reported that examines whether ME imparts a negative influence on otherwise healthy cells. The propagation of disease signaling is as plausible as the positive effects proposed above, and testing of this should remain a priority in the field.

## Author Contributions

PEBN and AO-M: concept, collection and assembly of data, manuscript writing. VAW: concept, financial support, manuscript writing.

## Conflict of Interest Statement

The authors declare that the research was conducted in the absence of any commercial or financial relationships that could be construed as a potential conflict of interest. The reviewer JM and handling Editor declared their shared affiliation.
